# Inflammatory biomarkers as predictors of systemic vs isolated pocket infection in patients undergoing transvenous lead extraction

**DOI:** 10.1016/j.hroo.2024.04.007

**Published:** 2024-04-15

**Authors:** Anne-Sophie Lacharite-Roberge, Sandeep Toomu, Omar Aldaas, Gordon Ho, Travis L. Pollema, Ulrika Birgersdotter-Green

**Affiliations:** ∗ Section of Cardiac Electrophysiology, Division of Cardiology, Department of Medicine, University of California San Diego, San Diego, California; † Division of Cardiovascular and Thoracic Surgery, University of California San Diego, San Diego, California

**Keywords:** cardiac device, cardiac implantable defibrillator, CIED extraction, CIED infection, inflammatory biomarkers, pacemaker

## Abstract

**Background:**

Cardiovascular implantable electronic device (CIED) infections are a common indication for device extraction. Early diagnosis and complete system removal are crucial to reduce morbidity and mortality. The lack of clear infectious symptoms makes the diagnosis of pocket infections challenging and may delay referral for extraction.

**Objective:**

We aimed to determine if inflammatory biomarkers can help diagnose CIED isolated pocket infection.

**Methods:**

We performed a retrospective analysis of all patients undergoing transvenous lead extraction for CIED infection at the University of California San Diego from 2012 to 2022 (N = 156). Patients were classified as systemic infection (n = 88) or isolated pocket infection (n = 68). Prospectively collected preoperative procalcitonin (PCT), C-reactive protein, and white blood cell count were compared between groups.

**Results:**

Pairwise comparisons revealed that the systemic infection group had a higher PCT than the control group (*P <* .001) and the pocket infection group (*P =* .009). However, there was no significant difference in PCT value between control subjects and isolated pocket infection subjects. Higher white blood cell count was only associated with systemic infection when compared with our control group (*P =* .018).

**Conclusion:**

In patients diagnosed with CIED infections requiring extraction, inflammatory biomarkers were not elevated in isolated pocket infection. Inflammatory markers are not predictive of the diagnosis of pocket infections, which ultimately requires a high level of clinical suspicion.

**Key Findings**

▪ History, physical examination, and clinical suspicion are the most reliable tools for the diagnosis of isolated cardiac device pocket infections.
▪ Isolated cardiac device pocket infections should be referred promptly to expert centers for consideration of transvenous or open lead extraction.
▪ Elevated inflammatory biomarkers do not correlate with a diagnosis of isolated cardiac device pocket infection and therefore cannot predict clinical outcomes.

## Introduction

Each year, the number of cardiovascular implantable electronic devices (CIEDs) increases with an aging population, technological advancements in electrophysiology and broader clinical indications. Worldwide, it is estimated that 1.7 million CIEDs are implanted yearly.^1^ Although CIEDs have become more reliable and have improved the quality of life and reduced mortality of several patient groups, infection remains a serious complication and a concern for clinicians in various subspecialties of medicine and surgery. According to the National CIED Infection Initiative put in place by the American Heart Association, as many as 1 in 20 patients with a CIED will experience an infection within 3 years of implantation.^2^ The reported estimated annual infection rate remains between 1% and 2%, and CIED infections are associated with significant morbidity, mortality and, elevated costs for healthcare systems across the nation.^3^^,^^4^

The recommendations from the Heart Rhythm Society are clear that complete device and lead extraction is crucial in eradicating CIED infection once the diagnosis is made.^5^ Additionally, extraction is time-sensitive and must be performed promptly to decrease mortality and morbidity related to CIED infection.^6^^,^^7^ The clinical presentation of CIED infection remains challenging, and we lack predictive tools to assist clinicians in making a diagnosis and initiating transfer to expert institutions. Isolated pocket infection can be particularly challenging, as patients often present with fewer symptoms and without clear infectious signs. To date, few studies have investigated the use of inflammatory biomarkers to assist in the early recognition and diagnosis of systemic CIED and isolated pocket infection. One small retrospective study by Lennerz and colleagues^8^ involving 25 patients revealed that the diagnosis of pocket infection is still largely based on clinical judgment and that C-reactive protein (CRP) and procalcitonin (PCT) with specific cutoffs may be an added data point to assist clinicians. In the current study, we present the largest patient cohort to date looking at inflammatory biomarkers in patients who underwent device and lead extraction. We aimed to determine if inflammatory biomarkers can help clinicians in the challenging diagnosis of isolated pocket infection.

## Methods

We performed a retrospective analysis of all patients undergoing transvenous lead extraction for CIED infection at the University of California San Diego between 2012 and 2022. Data were obtained from the electronic medical record (EMR) and included patient demographic, clinical, echocardiographic, and device characteristics such as device type, lead type, number of generator replacements, and initial indication for implantation. Regarding inflammatory biomarkers, we prospectively collected preoperative PCT, CRP, and white blood cell (WBC) count in all patients. Patients in our study group were classified as systemic infection or isolated pocket infection based on the presence or absence of sepsis. Patients in the control group underwent device extraction for noninfectious etiologies including lead fracture, device malfunction, or need for system upgrade. This study was approved by the Institutional Review Board of the University of California San Diego. The research reported in this manuscript has adhered the ethical principles of the Helsinki Declaration.

### Statistical analysis

Prospectively collected PCT, CRP, and WBC count were skewed and presented as median (interquartile range) and compared between groups using Kruskal-Wallis and Dunn-Bonferroni tests with *P* values adjusted for multiple comparisons. WBC count, CRP, and PCT were also used as categorial variables in the chi-square test to look at differences when 0, 1, 2, or 3 inflammatory biomarkers were abnormal. *P* values of <.05 were considered significant. All analyses were performed using IBM SPSS Statistics version 26.

## Results

All patients undergoing transvenous lead extraction for CIED infection at our institution from 2012 to 2022 were included in our study (n = 156). Baseline characteristics can be found in Table 1 and device and lead details can be found in Table 2. Our control group (n = 281) underwent CIED extraction for noninfectious etiologies such as lead fracture, lead perforation, device malfunction, severe chronic pain caused by the device, or need for system upgrade. Patients in the device infection group were classified as systemic infection (n = 88 [56%]) or isolated pocket infection (n = 68 [44%]) based on the presence or absence of sepsis. For patients to be placed into the systemic infection group, an EMR search was conducted, and sepsis had to be documented in the patient’s history and clinical presentation. All patients had 2 sets of blood cultures drawn prior to extraction. In the systemic infection group, 50 (57%) subjects had positive blood cultures for infectious pathogens, and in the isolated pocket infection group, 18 (26%) patients had positive blood cultures (Table 3). Vegetations were found on 43 (49%) device systems in the systemic infection group (Table 3). Subjects in the isolated pocket infection group had the following signs and symptoms documented in the EMR: warmth, erythema, fever, erosion, drainage, swelling, and pain. All patients with a suspected pocket infection had wound culture and lead cultures collected during extraction, in addition to 2 sets of blood cultures drawn prior to the procedure. In total, 66 (97%) of 68 patients with isolated pocket infections were found to have a positive device or pocket tissue culture for infectious pathogen (Table 3). Finally, 25% of patients with isolated pocket infection had positive lead cultures (Table 3).

**Table 1:** Baseline patient characteristics

	Systemic infection (n = 88)	Isolated pocket infection (n = 68)	Control (n = 281)
Age at the time of extraction, y	67 ± 11.0	65 ± 16.4	62 ± 16.0
Sex			
Female	22 (25)	12 (18)	94 (33)
Male	66 (75)	56 (82)	187 (67)
Body mass index, kg/m^2^	36 ± 29.0	34 ± 7.6	30 ± 27.1
Hypertension	69 (78)	42 (62)	160 (57)
Type 2 diabetes	52 (59)	19 (28)	66 (24)
Heart failure			
Ischemic cardiomyopathy	24 (27)	19 (28)	38 (14)
Nonischemic cardiomyopathy	18 (20)	16 (23)	80 (29)
Chronic renal insufficiency	36 (41)	18 (26)	39 (14)
End-stage renal disease on dialysis	15 (17)	5 (7)	4 (2)
Prior cerebral vascular accident	15 (17)	10 (15)	29 (11)
Chronic obstructive pulmonary disease	16 (18)	13 (19)	16 (6)
Obstructive sleep apnea	20 (23)	11 (16)	56 (20)
History of atrial fibrillation	53 (60)	28 (41)	95 (34)
Tobacco use at the time of extraction	6 (7)	2 (3)	15 (5)
Prior sternotomy	28 (32)	14 (21)	45 (16)
Medications at the time of extraction			
Coumadin	14 (16)	16 (24)	27 (10)
Direct-acting oral anticoagulant	38 (43)	12 (18)	59 (21)
Steroids	4 (5)	1 (1)	11 (4)
Insulin	27 (31)	11 (16)	26 (9)

Values are mean ± SD or n (%).

**Table 2:** Lead and device details

	Systemic infection (n = 88)	Isolated pocket infection (n = 68)	Control (n = 281)
Extracted lead type			
Pacemaker	53 (60)	37 (42)	145 (52)
Implantable cardioverter-defibrillator	34 (39)	23 (34)	120 (43)
Coronary sinus	11 (13)	6 (9)	47 (17)
Epicardial	0 (0)	2 (3)	0 (0)
Lead manufacturer			
Abbott	21 (24)	17 (25)	78 (28)
Biotronik	17 (19)	11 (16)	57 (20)
Boston Scientific	21 (24)	10 (15)	52 (18)
Medtronic	27 (31)	20 (29)	83 (30)
Other	2 (2)	10 (15)	11 (4)
Duration of implant, mo	67.52 ± 126.85	90.46 ± 165.84	100 ± 62.08
Patients with prior devices	31 (35)	37 (54)	134 (48)
Device changes per patient	1.38 ± 2.04	1.85 ± 1.44	1.44 ± 0.78
Was infection the indication for prior device changes?			
Yes	2 (6)	2 (5)	5 (2)
No	26 (84)	24 (65)	128 (96)
Unknown	3 (10)	11 (30)	1 (0.7)
Complete procedural success (all intended leads completely removed)	87 (99)	66 (97)	274 (98)
Wound closure type			
Sutured	64 (73)	12 (18)	281 (100)
Wound vacuum	24 (27)	52 (76)	0 (0)
Drain	0 (0)	3 (4)	0 (0)

Values are n (%) or mean ± SD.

**Table 3:** Inflammatory biomarkers and infection details

	Systemic infection	Isolated pocket infection
Inflammatory biomarkers		
White blood cell count (reference range and units: 4.0–10.0 1000/mm^3^)	9.0 ± 5.45	7.94 ± 4.20
Procalcitonin (reference range and units: 0.00–0.08 ng/mL)	3.97 ± 12.77	0.08 ± 0.09
C-reactive protein (reference range and units: <0.5 mg/dL)	10.73 ± 29.23	5.8 ± 14.41
Positive blood cultures	50 (57)	18 (26)
Positive device/pocket tissue culture	4 (5)	66 (97)
Positive lead culture	5 (6)	17 (25)
Vegetation on lead	43 (49)	8 (12)

Values are mean ± SD or n (%).

We found that PCT and WBC count medians differed significantly between the systemic infection, pocket infection, and control groups (*P <* .001 and *P =* .021, respectively). Details regarding inflammatory biomarker values in the study groups can be found in Table 3. Pairwise comparisons revealed that the systemic infection group had a higher PCT than the control group (*P <* .001) and the pocket infection group (*P =* .005). However, there was no significant difference in PCT value between the control group and the isolated pocket infection group (*P =* .512). Regarding pairwise differences in WBC counts, a higher value was also associated with systemic infection (*P <* .001) when compared with our control group and when compared with isolated pocket infection (*P <* .001). There was no significant increase in WBC counts when the pocket infection group was compared with the control group (*P =* .988). There was no significant difference in CRP levels between all 3 groups. When looking at the total number of abnormal laboratory results between PCT, WBC count, and CRP (0 of 3, 1 of 3, 2 of 3, or 3 of 3), the systemic infection group had significantly more laboratory abnormalities than the control group (*P <* .001). There was no significant difference between the systemic infection and isolated pocket infection group, or between the isolated pocket infection group and the control group (*P =* .163 and *P* = .555, respectively).

## Discussion

In the largest study to date analyzing inflammatory biomarker levels in patients diagnosed with CIED infection and undergoing complete system extraction, we demonstrated several findings that may further assist clinicians. The diagnosis of isolated pocket infection vs systemic infection remains a challenge, and a high degree of clinical suspicion, in addition to experience, is often required to pose a diagnosis.^8^, ^9^, ^10^ Close follow-up of patients reporting inflammatory symptoms localizing to the device pocket is key. Indeed, in our practice, the use of photographs to document progression of infection has proven incredibly useful. Prior studies have demonstrated that local inflammatory signs at the site of device implantation, despite a lack of systemic symptoms, are enough to diagnose CIED infection and proceed with referral to an expert center for extraction.^11^^,^^12^ Additionally, the most current guidelines recommend full system extraction for isolated pocket infection, even when blood cultures are negative, reiterating the importance of a prompt evaluation in the absence of systemic infection.^12^ One smaller study by Lennerz and colleagues^8^ also investigating inflammatory biomarkers in CIED infections showed that WBC count, CRP, and PCT are often normal in patients with isolated pocket infection. These observations are confirmed in our study and reaffirmed the need for a thorough physical examination and history taking at the time of presentation, as inflammatory biomarkers may not be accurate predictors of isolated pocket infections.

For several decades, leukocytosis has been an integral part of the systemic inflammatory response syndrome and the sepsis definitions.^13^ More recently, other biomarkers such as CRP and PCT have gained momentum in assisting with the diagnosis of infection and to guide antibiotic management. Studies have shown that PCT level is more sensitive and more specific than CRP level for differentiating bacterial infection from noninfectious causes of inflammation such as trauma, recent surgical intervention, or viral infections.^14^ A healthy adult generally will have a PCT value <0.05 ng/mL. A value between 0.05 and 0.5 ng/mL is interpreted as unlikely systemic infection, although localized infection is possible. Systemic infection becomes likely when the PCT value ranges from 2 to 10 ng/mL.^15^, ^16^, ^17^ In our isolated pocket infection group, the highest recorded PCT value was 0.41 ng/mL, and the mean was 0.08 ng/mL (Table 3). The localized nature of the infection could be responsible for these findings and for the lack of significant difference in PCT level with our noninfectious control group. As previously suggested in smaller studies, establishing a lower cutoff value or detection limit specifically for localized infection may warrant further investigation and could be of value in the case of isolated pocket infections.^8^

When collecting and analyzing PCT levels in isolated pocket infection patients, one must consider PCT’s rapid decline when antibiotics are initiated. Several studies and meta-analyses were published regarding PCT-guided strategies for treatment of patients with various systemic infections.^18^, ^19^, ^20^ In our isolated pocket infection group, 71% of patients were treated with antibiotics preoperatively, and it is unknown if PCT was collected before or after antibiotics were administered. As one would expect PCT to be lower in isolated infection than in systemic infection, if levels were drawn following antibiotics initiation, this may have contributed to a lack of significant difference with our noninfectious control group. Finally, although PCT has shown promise in assisting with the diagnosis and management of sepsis, its considerable cost has motivated the publication of cost-effectiveness analyses.^21^ Given the lack of PCT elevation in patients with isolated pocket infection using the current known cutoffs, it may not be cost-effective to check PCT in all patients with suspected CIED infections if the physical examination is already suggestive of localized infection. This is keeping in mind that the average cost of an admission for a single CIED infection, which was estimated at $146,000 in 2008.^22^

As predicted, there was a significant difference in WBC count in patients with CIED infection presenting with sepsis, as compared with our noninfectious group. However, there was no significant difference with the isolated pocket infection group. This is a notable finding, as leukocytosis remains the most historically trusted laboratory finding for diagnosis of infection. Consequently, in the case of isolated pocket infection, the diagnosis of localized infection should not be automatically ruled out if the WBC count is within normal limits.

Interestingly, a higher percentage of patients with isolated pocket infection had positive lead culture than patients with systemic infection. However, there was no statistically significant correlation between positive inflammatory biomarkers positive and patients with positive lead culture (*P =* .92).

### Limitations

Our study was retrospective with a relatively small sample size, which limited our analyses and ability to perform multivariate statistical tests. Additionally, few patients in both the systemic infection and in the isolated pocket infection groups did not have a CRP or PCT collected prior to extraction, which limited our ability to draw conclusions. As previously noted, it would be beneficial to note the time of PCT collection and whether it was collected before or after antibiotic treatment, given the known rapid effect of antibiotics treatment on PCT value.

## Conclusion

The diagnosis of CIED infection remains challenging, and our findings reiterate the importance of a thorough in person patient evaluation, especially for early and isolated pocket infections. Although inflammatory biomarkers such as WBC count, PCT, and CRP remain important adjunct diagnostic tools, we have yet to find a biomarker that can reliably rule out and predict isolated pocket infection. Our findings indicate that inflammatory biomarkers do not correlate with clinical outcomes in regard to isolated pocket infection. This conclusion is particularly important when evaluating patients with subtle clinical signs or symptoms of pocket infection, as guidelines would still recommend CIED extraction in the absence of positive blood cultures. Consequently, there should be no hesitation or delay to refer patients to expert extraction centers when clinical suspicion is high but laboratory results are within normal limits.
